# A Short Review of Research Progress on the Synthesis Approaches of Aza-Dibenzocyclooctyne Derivatives

**DOI:** 10.3390/molecules28093715

**Published:** 2023-04-25

**Authors:** Yinming He, Li Liu, Liang Cheng

**Affiliations:** 1 Beijing National Laboratory for Molecular Sciences (BNLMS), CAS Key Laboratory of Molecular Recognition and Function, CAS Research/Education Center for Excellence in Molecular Sciences, Institute of Chemistry, Chinese Academy of Sciences, Beijing 100190, China; 2 University of Chinese Academy of Sciences, Beijing 100049, China

**Keywords:** aza-dibenzocyclooctyne, biarylazacyclooctynone, synthesis, bioorthogonal reaction, strain-promoted azide-alkyne cycloaddition

## Abstract

Cyclooctyne molecules have found wide applications in the strain-promoted azide–alkyne cycloaddition (SPAAC) reactions, which avoid the biotoxicity caused by the use of Cu(I) catalysts. Among the various cyclooctyne systems, dibenzocyclooctyne (DBCO) series have displayed the highest reaction activity. However, the synthesis processes of such structures are time-consuming, which to some extent limit their large-scale development and application. This review has summarized current synthesis routes of two DBCO molecules, aza-dibenzocyclooctyne (DIBAC) and biarylazacyclooctynone (BARAC).

## 1. Introduction

Bioorthogonal chemistry has emerged as a rapidly expanding field over the past few decades, driven by the need to study and manipulate biomolecules in their natural environment. It refers to the chemical transformations that can occur within living systems without perturbing natural biological processes, enabling researchers to label, detect, and manipulate biomolecules with high specificity and efficiency [1,2,3]. This approach has revolutionized many areas of biology and medicine, from basic research to drug discovery and diagnostics [4,5,6,7,8].

One of the most widely used bioorthogonal reactions is the Cu(I)-catalyzed azide–alkyne cycloaddition (CuAAC), which enables efficient and selective conjugation of azide and alkyne functionalized molecules [9]. This reaction has been used to label and track biomolecules, such as proteins and nucleic acids, in live cells and animals, and to image biological processes in real-time. Other bioorthogonal reactions include tetrazine ligation, [10,11] strain-promoted alkyne–nitrone cycloaddition (SPANC), [12] and photo-triggered reactions, [13] among others.

Recent advancements in bioorthogonal chemistry have focused on the development of new bioorthogonal reactions and probes that can be used for specific applications [14]. One of the most promising new bioorthogonal reactions is the inverse-electron-demand Diels–Alder (IEDDA) reaction, [15] which can be used for site-specific labeling of proteins and nucleic acids in live cells. Another area of active research in bioorthogonal chemistry is the development of new bioorthogonal probes that can be used for imaging and detection of biological molecules and processes [16]. These probes can be designed to target specific biomolecules or pathways, and can be used for both in vitro and in vivo imaging. For example, activatable probes have been developed that are only activated upon binding to a target biomolecule, allowing for highly specific and sensitive detection. Additionally, probes that emit light in response to biological stimuli, such as pH or redox potential, have been developed for in vivo imaging [17,18].

**Figure 1:**
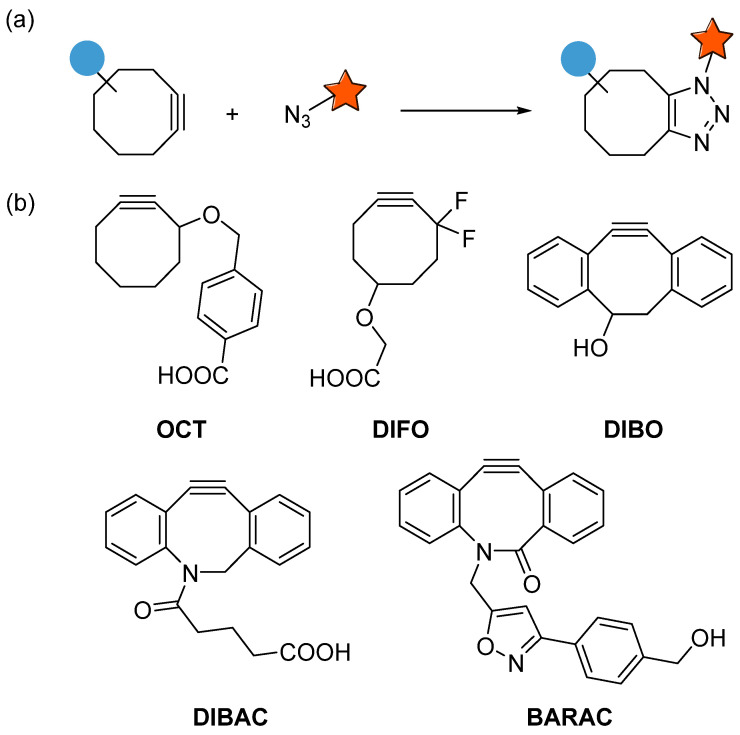
(**a**) Strain-promoted azide–alkyne cycloaddition. (**b**) Selected cyclooctyne molecules applied in SPAAC.

The 1,3-dipolar cycloaddition reaction between azides and alkynes is perhaps one of the most commonly used bioorthogonal reactions [19,20]. It allows for the formation of 1,2,3-triazoles, which have a wide range of applications in chemical biology [21]. Traditionally, this type of reaction required high temperatures and pressures. However, in 2001, Sharpless, Meldal and colleagues reported the development of copper(I)-catalyzed azide–alkyne cycloaddition (CuAAC), which could be carried out under mild conditions at room temperature [9,22]. This breakthrough led to the widespread use of click chemistry, a term used to describe the reaction’s simplicity and efficiency. Although CuAAC is widely used in bioorthogonal chemistry, the cytotoxicity of copper(I) limits its use in biological systems. As a result, researchers have focused on developing copper-free alternatives. As early as in 1961, Wittig and Krebs noticed that cyclooctyne and azidobenzene could react by strain promotion to form triazole [23]. In 2004, Bertozzi and colleagues revisited this reaction and developed the first cyclooctyne OCT for copper-free click chemistry [7]. This type of reaction is known as strain-promoted azide–alkyne cycloaddition (SPAAC) (Figure 1).

SPAAC has become an essential tool in chemical biology because it can be used in live cells and in vivo. One of the benefits of SPAAC is its compatibility with a wide range of functional groups. The reaction’s rate can also be increased by modifying the cyclooctyne’s structure, which allows for the development of highly selective and efficient reactions (Table 1). In recent years, researchers have continued to develop and refine SPAAC. For example, cyclooctynes with different strain-promoting groups have been developed to increase the reaction rate. The use of SPAAC has also expanded to include applications in drug discovery and materials science. For example, SPAAC has been used to develop drug conjugates and to create polymer networks.

Compared with terminal alkynes, the reaction rate of OCT increases only slightly, and its water solubility is not good enough. Bertozzi improved cyclooctyne by introducing a fluorine atom with an electron-withdrawing effect at the propargylic position, resulting in DIFO (difluorinated cyclooctyne), which significantly increased the reaction rate with azides [24,25]. Subsequently, Boons reported DIBO (dibenzocyclooctyne), in which the dibenzene system can increase strain energy, making it significantly active [26,27,28]. In 2010, van Delft introduced a nitrogen atom into cyclooctyne to obtain DIBAC (aza-dibenzocyclooctyne) [8,29]. In the same year, Bertozzi et al. optimized cyclooctyne again by introducing the amide structure into the ring to obtain BARAC (biarylazacyclooctynone) [30]. These compounds are among the most reactive in SPAAC (Table 1). However, the synthesis of these compounds is time-consuming and always require harsh conditions, which limits their application. Considering that there has not yet been a comprehensive review in this area, herein we will briefly summarize the current synthesis approaches for the DIBAC and BARAC series.

## 2. Synthesis Approaches for DIBAC

**Figure 2:**
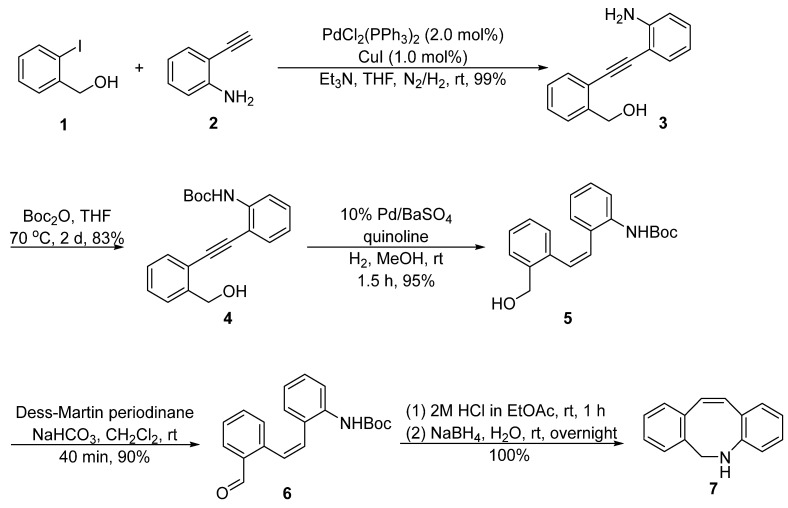
Synthetic approach of intermediate **7** by van Delft.

DIBAC was initially developed by van Delft, [8] who used 2-iodobenzyl alcohol **1** and 2-ethynylaniline **2** as the starting materials (Figure 2). A Sonogashira coupling reaction was performed to construct the internal alkyne **3**, followed by the protection of the aniline and semi-hydrogenation catalyzed by Pd/BaSO_4_ to deliver the *cis*-alkene **5**. The terminal hydroxyl methyl was oxidized to aldehyde using the Dess–Martin oxidation reaction and then passed through a reductive amination reaction with the intramolecular amino group to obtain the key intermediate **7**. The synthetic route contained multiple steps, however, each step exhibited a relatively high yield, and the total yield for preparing intermediate **7** can reach as high as 70%.

**Figure 3:**
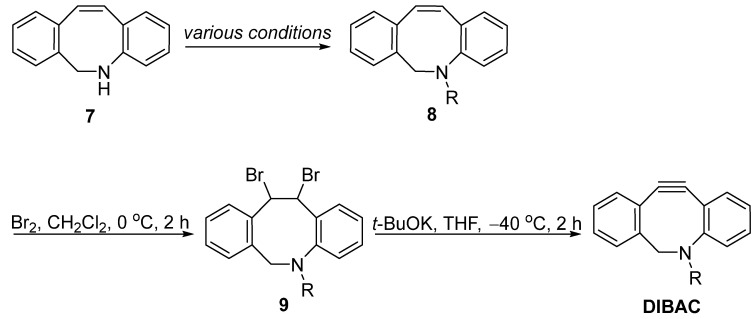
Synthesis of DIBAC from intermediate **7** through bromination and elimination.

With the key intermediate **7** in hand, the most commonly used method for synthesizing DIBAC could be achieved in three steps (Figure 3). A simple alkylation would introduce functional probes (azide, alkyne, biotin, etc.) onto the nitrogen under various conditions. Following this, bromination and base-prompted elimination would then give the corresponding product, DIBAC. 

**Figure 4:**
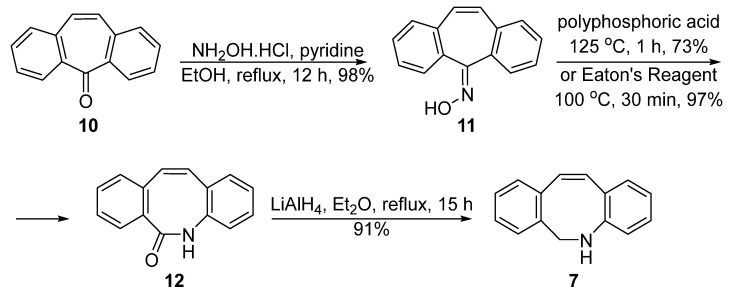
Popik’s improvement of the synthesis of DIBAC.

Compared with van Delft’s route, Popik et al. developed another synthesis route with fewer steps (Figure 4) [31]. Dibenzosuberone **10**, a commonly fused tricyclic framework, was used as the starting material. It was converted to oxime **11** and then treated by polyphosphoric acid, which functioned both as an acid catalyst and a dehydrating agent, at 125 °C for 1 h to afford the amide **12** in 97% yield. A selective reduction in the amide linkage with lithium aluminum hydride would then deliver the expected amine **7** in 91% yield. Although there were fewer steps in Popik’s pathway, the overall yield was not superior (40% for three steps from **10**). However, the utilization of cheap reagents (hydroxylamine and polyphosphoric acid) and easy operation without low temperature experiments made it more appealing. 

The key step in the Popik’s route is the Beckmann rearrangement, which was promoted by polyphosphoric acid under a high temperature. Adronov et al. believed that the poor solubility of the polyphosphoric acid may be the reason for the low yield of this step, and therefore used the Eaton reagent instead for this key rearrangement [32]. It turned out that the 10 wt% of phosphorus pentoxide solution in methanesulfonic acid [33] is an excellent alternative to polyphosphoric acid for the nitrilium ion formation, with which the intermediate **7** could be obtained in 90% yield, even in gram-scale processes (Figure 4). Only one chromatography purification step was needed in this modified DIBAC synthesis process, which greatly simplified the whole process. 

**Figure 5:**
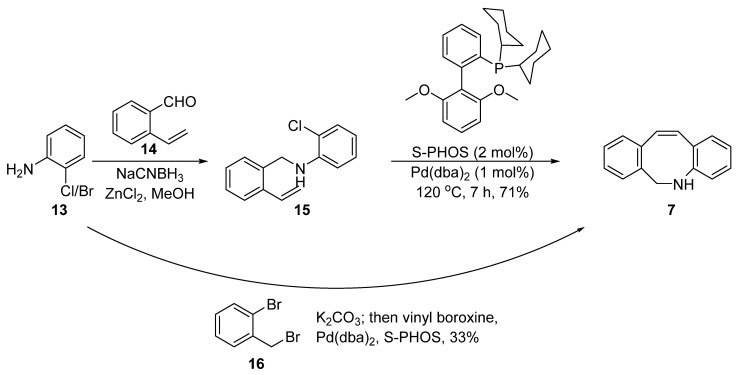
Synthesis of intermediate **7** via Suzuki–Heck coupling.

In 2013, Schubert et al. reported a different method for constructing the DIBAC skeleton using Pd-catalyzed cyclization (Figure 5) [34]. This method relied on the regioselectivity of the intramolecular Heck reaction between 2-vinylbenzaldehyde **14** and 2-bromoaniline **13**. The former intermediate was prepared via a vinylation of bromo-benzaldehyde by Suzuki coupling with vinylboroxine [35] or via the Hiyama reaction with vinylsiloxane [36]. A subsequent reductive imine condensation with the 2-chloroaniline **13** afforded the *N*-benzylaniline **15** containing a vinyl and halide moiety, which then underwent an intramolecular Suzuki–Heck coupling to obtain the key intermediate **7**. The reaction conditions (phosphine ligand, aryl halide, and temperature) were carefully optimized to suppress side reactions and the anticipated product **7** was isolated in 73% (two steps) when Pd(dba)_2_ and S-PHOS was applied. 

Considering the modular nature of this method, Schubert further extended it to a one-pot synthesis (Figure 5). They used commercially available 2-bromo-benzyl bromide **16** and 2-chloroaniline **13** as the starting materials. The mixture was subjected to the standard intramolecular amination and Suzuki–Heck coupling and the final product **7** was obtained in 33% yield. The yield was a little lower than that of the two step route due to unavoidable double alkylation of the amino group, and thus further research and optimization may be required. 

**Figure 6:**
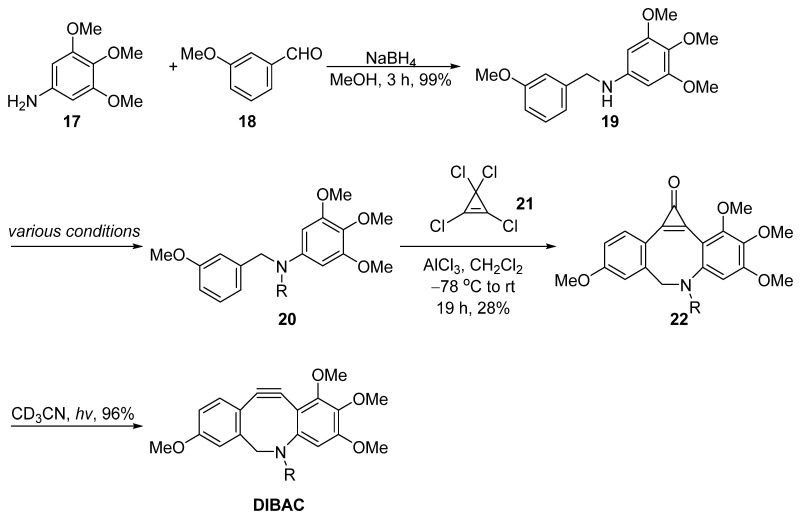
Synthesis of DIBAC by Pietzsch.

Unlike the strategies mentioned above that involved dibromination and dehydrohalogenation to generate the triple bond within the dibenzosuberane, Pietzsch et al. utilized ultraviolet irradiation to promote the elimination of carbon monoxide from the 2-cyclopropen-1-one motif, resulting in the release of the C–C triple bond (Figure 6) [37]. The key step was the ring closure with tetrachlorocyclopropene **21**. Starting from 3,4,5-trimethoxyaniline **17** and 3-methoxybenzaldehyde **18**, a reductive amination delivered the *N*-benzylaniline **19**, followed by a dual Friedel–Crafts reaction with tetrachlorocyclopropene **21** to produce the key intermediate **22**. The 2-cyclopropen-1-one motif was highly unstable under ultraviolet light irradiation, which then led to the decomposition of the cyclopropene with the release of carbon monoxide and the formation of the corresponding triple bond. This method was simple, rapid, started from inexpensive materials and delivered the final product in a three-step synthesis. Although the overall yield was low (23%), further improvements may be explored, such as the Friedel–Crafts acylation efficiency.

**Figure 7:**
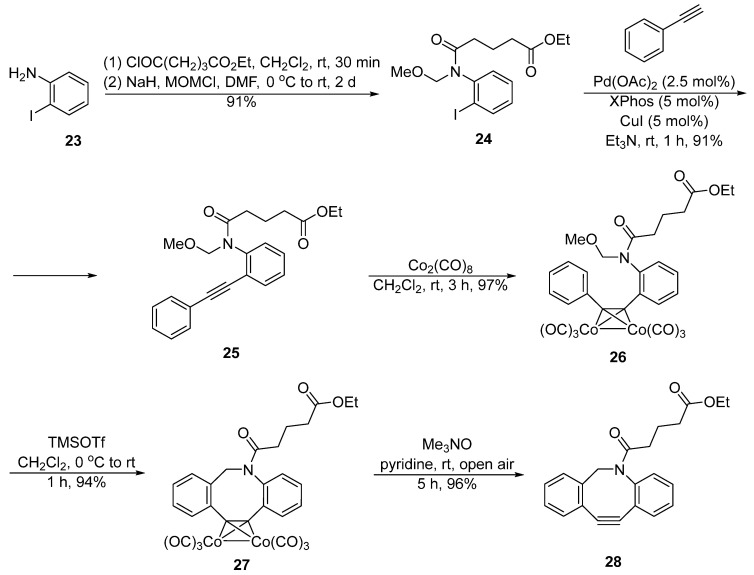
Synthesis of DIBAC by Hosoya.

While van Delft’s method has been commonly used for the synthesis of DIBAC, recently Hosoya et al. designed new alkyne−cobalt complexes to prepare DIBAC and its derivatives (Figure 7) [38]. This strategy is based on the utilization of Pictet−Spengler reaction, in which *N*-methoxymethyl amide **24** was generated from *o*-iodoaniline **23** through amidation and *N*-methoxymethylation. A Sonogashira coupling reaction was performed to construct diarylacetylene **25** that was later treated with Co_2_(CO)_8_ to yield an alkyne−cobalt complex **26**. The Pictet−Spengler reaction was applied to afford the DIBAC−cobalt complex **27**. Finally, the cobalt complex **27** was disassociated using the Me_3_NO/pyridine/air system to deliver the DIBAC derivative **28**. The overall yield was 72% (six steps), which is higher than van Delft’s synthesis method.

## 3. Synthesis Approaches for BARAC

**Figure 8:**
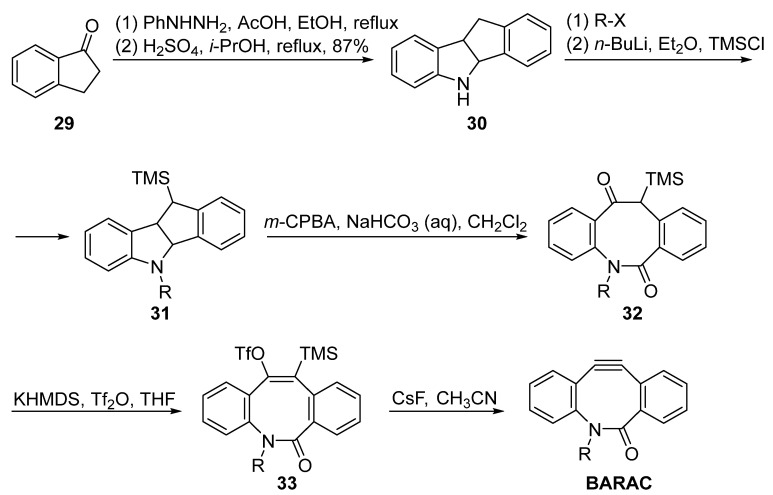
Synthesis of BARAC by Bertozzi.

BARAC is another useful cyclooctyne probe, which displays a constant reaction rate higher than that of DIBAC. However, its synthesis methods are quite limited. Bertozzi et al. subjected the Fischer indole intermediate from 1-indanone **29** and phenylhydrazine to an acid-promoted rearrangement to obtain the eight-membered skeleton **30** (Figure 8). The key intermediate was then alkylated with the corresponding linkage and installed in the TMS group as a placeholder. The decorated **31** was subjected to oxidative cleavage with *m*-CPBA and then converted to the triple bond precursor TMS substituted vinyl trifluoromethanesulfonate **33**, which was then cleaved using fluoride ion to generate the BARAC. 

**Figure 9:**
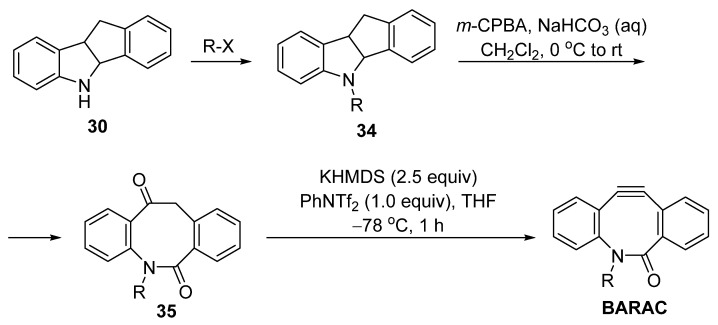
Okano’s optimization for the synthesis of BARAC.

In 2019, Okano et al. improved Bertozzi’s synthesis method (Figure 9) [39]. Instead of introducing a TMS group onto **34**, the eight-membered skeleton was firstly subjected to an oxidative cleavage of an amide–ketone **35**. Using the KHMDS and PhNTf_2_ as the dehydrogenation combination, the target BARAC product was obtained in good yields (74% when R = Me). However, when the final step was conducted at 0 °C or higher, only trace amount of the product was observed. 

**Figure 10:**
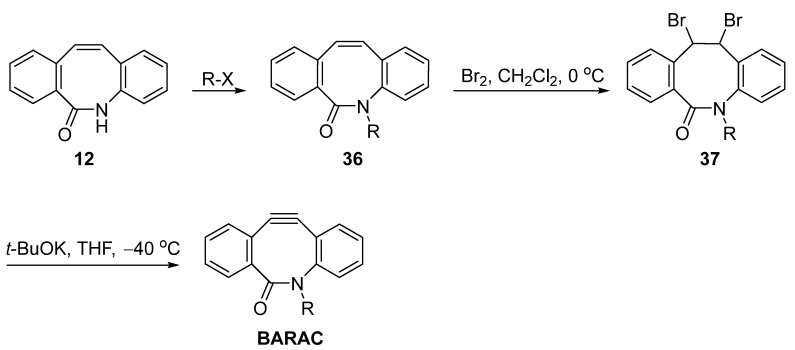
Adronov’s synthetic route via the amide to BARAC.

As mentioned before, an amide intermediate **12** was generated during the synthesis of DIBAC via the Beckmann rearrangement (Figure 4). Therefore, Adronov et al. considered using this process to prepare BARAC derivatives (Figure 10) [32]. Similar to the synthesis method of DIBAC, they first prepared the alkylated amide and then subjected the **36** to dibromination to obtain **37**, followed by base-facilitated elimination. However, the corresponding BARAC was generated in less than 10% (NMR and TLC) and could only be trapped by click reaction with benzyl azide. Therefore, further optimization was still required. 

## 4. Conclusions

In conclusion, we have summarized current synthetic methods of dibenzocyclooctyne (DBCO), i.e., the DIBAC and BARAC derivatives. Both of these probes are widely used in SPAAC reactions, but current synthetic routes still display some drawbacks, such as long synthetic routes, expensive starting materials and catalysts, harsh reaction conditions, and low yields, which makes it difficult to produce them on a large scale or generate a diverse library of derivatives. Therefore, there is still a lot of room for improvement in these synthetic aspects. Additionally, of course, with the advancements of bioorthogonal chemistry in the expansion of toolkits for studying complex biological systems, new structures and efficient approaches may also emerge in the future. We anticipate that in the near future, new structures based on DBCO will continue to be discovered, and their synthetic methods will also become more efficient. We hope that this mini review can bring some inspiration to those who are devoted to this area.

## Figures and Tables

**Table 1:** Second-order reaction rate constants for different cyclooctyne systems with benzyl azide at room temperature.

DBCO Compound	Solvent	*k* (10^−3^ M^−1^ s^−1^)
OCT	CD_3_CN	2.4
DIFO DIBO	CD_3_CN CH_3_OH	76 57
DIBAC BARAC	CD_3_OD CD_3_CN	310 960

## Data Availability

Not applicable.
